# Incidence and impact of involuntary rib fracture after intercostal spreading for thoracotomy

**DOI:** 10.1007/s13304-024-01929-w

**Published:** 2024-07-27

**Authors:** Sebastiano Angelo Bastone, Emanuela Re Cecconi, Alexandro Patirelis, Vincenzo Ambrogi

**Affiliations:** 1 Department of Thoracic Surgery, Tor Vergata University Polyclinic, Viale Oxford, 81, 00133 Rome, Italy; 2 Ph.D. Program in Applied Medical-Surgical Sciences, Department of Surgical Sciences, Tor Vergata University Polyclinic, Rome, Italy

**Keywords:** Rib fracture, Intercostal spreading, Rib spreading, Thoracotomy, Thoracic surgery

## Abstract

Thoracotomy with rib spreading still remains the preferred approach for complex surgery. Rib fracture is considered a frequent involuntary event during this approach, but its real incidence has not been adequately investigated yet. In this study, we evaluated the incidence of rib fracture after thoracotomy, the possible risk factors and the relationship with post-operative pain and complications. We retrospectively analyzed the medical records of single-institution patients submitted to lateral thoracotomy from January 2016 to June 2023. Exclusion criteria were traumatic etiology and a medical history of osteoporosis. The presence of rib fracture was retrieved by surgical reports or post-operative chest X-ray. Basal and evoked pain after surgery was assessed by Visual Analogue Scale. The considered 30-day post-operative complications were atelectasis, need of endoscopic broncho-aspiration, pneumonia and pleural effusion. A total of 367 consecutive patients underwent thoracotomy in the study period. The median age was 68 (interquartile range 60–75) years. Rib fracture was detected in 179/367 (48.8%) patients. Incidence did not significantly vary throughout years (*p* = 0.98). The risk of developing post-thoracotomy rib fractures was significantly associated with age greater than the median value (*p* = 0.003). The presence of rib fracture was related to significantly more elevated evoked pain at 48 h after surgery (*p* = 0.039) and a higher incidence of complications (32/179 vs 20/188; *p* = 0.047). Our study demonstrated that rib fracture occurs in almost half of the thoracotomies. Older patients are more likely to develop this event, which significantly correlates to increased evoked post-operative pain and higher rate of post-operative complications.

## Introduction

Rib fractures are usually considered an irrelevant event after intercostal spreading during thoracic surgery. Nevertheless, this occurrence may provoke more pain than that triggered after a simple compression of the intercostal bundle, with a relevant postoperative impact on ventilatory dynamics [1–3].

In spite of the potential impact on post-operative recovery, the incidence of rib fracture after thoracotomy is usually considered negligible. Notably, we did not find information about this data when reviewing the literature. The outcomes of rib fractures are almost exclusively studied in patients with blunt traumatic injuries [1, 2], including in this amount also iatrogenic fractures after cardio-pulmonary resuscitation [3, 4]. As a matter of fact, the incidence and the outcome of post-spreading fractures is a nearly non-investigated field.

In this retrospective study, we have firstly evaluated the incidence of involuntary iatrogenic rib fracture after thoracotomy with costal spreading for elective thoracic surgery. As second endpoint, we have investigated those clinical factors which may be most correlated to this event and its relationship with post-operative pain and complications.

## Methods

This is a retrospective and monocentric study focused on the incidence of involuntary rib fractures in patients undergoing open thoracotomy. The study sample consists of consecutive patients submitted to rib spreading with mechanical devices accrued from January 2016 to June 2023. Patients’ data were retrieved by clinical records and stored in a dedicated database with the permission of our Internal Review Board. Collected data were demographics (i.e., sex and age), type of surgery (i.e., lung resections, pleural, mediastinal and diaphragmatic surgery), post-operative pain levels and surgery related complications developed within 30 days from the operation. Inclusion criteria were age over 18 and being subjected to thoracotomy either ab initio or after conversion during video-thoracoscopic procedure with positioning of a rib spreader (i.e., Finochietto–Burford spreader). Thoracotomies performed after traumatic events were excluded because probable pre-existent rib fractures. Diagnosis of osteoporosis or the presence of other medical conditions related to an alteration of bone density (i.e. corticosteroid therapy, endocrine diseases like uncontrolled hyperthyroidism or chronic inflammatory diseases) was an exclusion criterion due to the obvious bone frailty. Nevertheless, this number was limited, and it did not significantly affect incidence rate of fracture.

The event of post-spreading rib fractures was ascertained by retrieving surgical reports. Indeed, it is our policy to describe this event whenever evident during the procedure. In addition, incidental fractures neglected at the operative notes were reassessed by reviewing post-operative day-1 chest X-Ray. The number of post-procedural rib fractures was also considered and reported in a specific database field.

### Surgical details

All procedures were performed through lateral thoracotomy with the patient lying on the opposite side. The incision was always performed at the 5th intercostal space and the *latissimus dorsi* muscle was identified, dissected along its anterior margin and entirely spared. This dissection allowed wide posterior retraction permitting the satisfactory view of approximately 20 cm of the *serratus anterior* muscle. The latter one was separated along its digitations avoiding muscle cutting as much as possible. After having evidenced the chest wall, the inferior rib of the target intercostal space was individuated and dissected. The parietal pleura was evidenced and opened along the rib margin for an approximate length of 20 cm. Once adequately opened the pleural space and freed the incidental pleuro-pulmonary adhesions, a Finochietto–Burford rib spreader was positioned. The spreader was progressively opened till reaching the distance between ribs to allow the entrance of at least one hand (approximately 10 cm of maximal displacement).

At the end of surgery, two chest wall drainages were placed, with the anterior one draining the pleural dome and the posterior one oriented towards the basis.

Costal repair was always performed when fractures were evident during surgery and when the extremities of the broken rib were not reducible, using an X-shaped stitch of polyglactin 910 2 suture embracing the two stumps. All patients, regardless of the presence of rib fracture, were submitted to a serratus anterior plane block with ropivacaine by our anesthesiology team.

### Post-operative work-up

Basal pain at 24 and 48 h from the operation was assessed by Visual Analogue Scale (VAS) [5] and collected. 48-h after surgery evoked pain after coughing was also investigated. We decided not to evaluate 12-h post-operative pain due to possible residual effects of anesthetics, which could have altered real pain perception by the patient.

Post-operative analgesic therapy was the same for all patients. It was based on acetaminophen 1 g i.v. three times per day and tramadol 200 mg i.v. per day under continuous administration.

All patients received physiotherapy at post-operative day-1 consisting in early mobilization and respiratory exercises under a dedicated physiotherapist. As above stated, chest X-ray was performed in all instances at post-operative day-1.

Considered 30-day post-operative complications related to rib fractures were atelectasis, need of endoscopic broncho-aspiration, pneumonia, and pleural effusion.

### Statistical analysis

Statistical analysis was performed using SPSS (IBM Corp. Released 2016. IBM SPSS Statistics, Version 26.0; Armonk, NY, USA: IBM Corp.). *P*-value less than 0.050 was considered statistically significant. Continuous variables were reported as median with interquartile range (IQR) due to non-normal distribution. Categorical ones were presented as frequencies. Histograms were used to display some categorical data.

Descriptive analysis was carried out with non-parametric Mann–Whitney *U* test for continuous variables and Pearson’s *χ*^2^ for categorical ones.

We evaluated possible predictors of rib fractures after intercostal spreading through a binary logistic regression including the following factors: sex and age. Continuous variables were dichotomized according to the median value. Factors significantly affecting the risk of rib fractures at univariable analysis were included in a multivariable one.

## Results

The overall original group entailed 421 consecutive patients undergoing thoracotomies. Out of this amount, 26 patients having traumatic etiology and another 17 patients with a clear diagnosis of osteoporosis were excluded. Eventually, 11 patients were not included for incomplete data. Thus, the total number of the sample reduced to 367 individuals. Main demographic and clinical data are summarized in Table 1. Median age was 68 years (IQR 60–75) and 69.2% of them were male.

**Table 1:** Patients undergoing thoracotomy

Variable	Intact ribs (*n* = 188)	Fractured ribs (*n* = 179)	*p*-value
Age, median (IQR)	65 (59–73.75)	70 (61–76)	**0.011**
Median age (68 years), *n* (%)			**0.003**
< 68	105 (55.9)	72 (40.2)	
≥ 68	83 (44.1)	107 (59.8)	
Sex, *n* (%)			0.12
Male	137 (72.9)	117 (65.4)	
Female	51 (27.1)	62 (34.6)	
Type of surgery, *n* (%)			0.73
Sublobar lung resection	49 (26.1)	38 (21.2)	
Lobectomy	77 (40.9)	81 (45.2)	
Pneumonectomy	12 (6.4)	17 (9.5)	
Pleural decortication	39 (20.7)	32 (17.9)	
Mediastinal surgery	8 (4.3)	8 (4.5)	
Diaphragm surgery	3 (1.6)	3 (1.7)	
Post-operative complication, *n* (%)			**0.047**
No	168 (89.4)	147 (82.1)	
Yes	20 (10.6)	32 (17.9)	

Significant *p*-values are reported in bold

Distribution of demographic and clinical details for intact and fractured ribs. Continuous data are expressed as median (interquartile range [IQR])

**Fig. 1:**
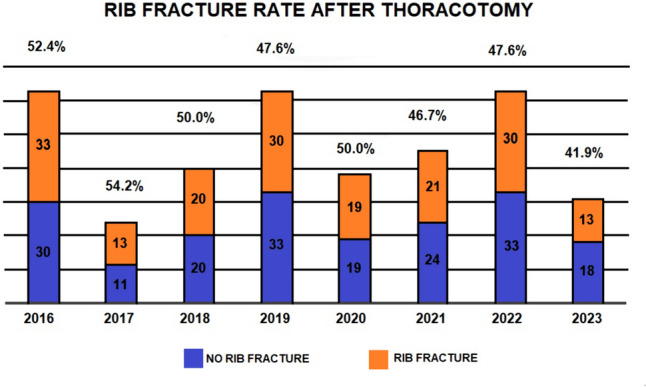
Incidence of rib fracture after thoracotomy from 2016 to 2023

Out of this number, we documented at least one involuntary rib fracture in 179 of patients (48.8%) that is almost one half of the patients undergoing thoracotomy. Among them, a vast majority, equal to 72.1% (129/179), presented fractures of two adjacent ribs. Percentages of rib fractures did not significantly vary throughout years (*p* = 0.98), with 2023 presenting the lowest incidence so far (Fig. 1). In 12.8% of cases (23/179) the same rib was broken in two separate areas.

The risk of developing post-thoracotomy rib fractures was significantly (*p* = 0.003) associated with the dichotomized age (median age, 68 years old) at univariable analysis. As expected, the risk was higher in older patients, with an odds ratio of 1.9 (95% CI 1.2–2.8). This was the only significant demographic predictor of rib fracture. Despite the general belief, female sex was not significantly correlated with a higher risk of rib fractures (*p* = 0.12) although the relative percentage of women was higher: 34.6% (62/179) vs 27.1% (51/188). Given the unicity of significant predisposing factor, multivariable analysis was unfeasible.

Basal VAS scores at 24 and at 48 h did not differ statistically in patients with and without fractures. On the other hand, evoked pain at 48 h was higher in patients with rib fractures (*p* = 0.039) (Table 2). The presence of two adjacent rib fractures did not show a higher pain level compared to single one, although evoked pain at 48 h after surgery resulted quasi-significant (*p* = 0.056).

**Table 2:** Post-operative pain evaluation according to visual analogue scale

Variable	Intact ribs (*n* = 188)	Rib fractures (*n* = 179)	*p*-value	Single rib fracture (*n* = 50)	Two ribs fracture (*n* = 129)	*p* value
24-h post-operative pain	4 (4–5)	4 (4–5)	0.30	4 (3–5)	4 (4–5)	0.20
48-h post-operative pain	4 (4–4)	4 (4–5)	0.11	4 (3.7–4)	4 (4–5)	0.21
48-h post-operative evoked pain	6 (5–6.75)	6 (5–7)	**0.039**	6 (5–7)	6 (5–7)	0.056

Significant *p*-values are reported in bold

Data are expressed as median (interquartile range)

The rib fractured group showed a significant higher rate (*p* = 0.047) of 30-day post-operative complications compared to the other one: 17.9% (32/179) vs 10.6% (20/188). As reported, the total amount of complications was 39 for rib fractured group and 25 for the intact ribs group (Table 3). Four patients in intact ribs group and 6 in the rib fractured group presented more than one complication.

**Table 3:** Post-operative complications

Complications	Intact ribs (*n* pts = 20)^a^	Rib fractures (*n* pts = 32)		Intact vs fractured *p* value
		Single rib (*n* pts = 6)^b^	Two ribs (*n* pts = 26)^c^	
Pleural effusion	7 (28.0%)	3 (37.5%)	9 (29.0%)	0.85
Atelectasis	10 (40.0%)	3 (37.5%)	13 (41.9%)	1.00
Need of broncho-aspiration	5 (20.0%)	1 (12.5%)	6 (19.4%)	0.79
Pneumonia	3 (12.0%)	1 (12.5%)	3 (9.7%)	0.80
Patients with complications	20/188 (10.6%)	32/179 (17.9%)		**0.047**

Significant *p*-values are reported in bold

^a^Four patients with more than one complication

^b^Two patients with more than one complication

^c^Four patients with more than one complication

## Discussion

The increasing popularity of mini-invasive accesses made thoracotomy with rib spreading progressively infrequent. Indeed, the advantages of small incisions are self-evident and recognized, while offering an equally comparable therapeutic outcome [6].

However, open approaches still remain the preferable way when dealing with complex operations, unfavorable anatomy, obliterated pleural spaces or intraoperative complications [7–9]. Despite the pursued policy of our department to privilege minimally invasive procedures, we experienced an almost uncompressible percentage of thoracotomies in the last decade.

A probable complication of rib spreading during thoracotomy is accidental rib fracture. A recent experimental study by Chanoit et al. has demonstrated how thoracotomy is a traumatic procedure, reaching forces from 165 to 470 N to achieve an adequate exposition of the anatomic structures [10]. In spite of the concrete discomfort represented by rib fractures, this event has not been worthy of a specific finalized investigation yet. During the work-up preliminary to the present study, we were able to retrieve only few and long-standing papers about the presence of rib fractures after sternotomy [11, 12]. Notably, we did not find any review specifically aimed at describing the incidence of rib fractures after intercostal spreading during thoracotomies.

According to our results, almost one half of the patients submitted to thoracotomy presented at least one rib fracture. Interestingly, the trend remained fairly constant over the years, ranging from 41.9 to 54.2%. Nowadays, these values cannot be considered acceptable for both patients and surgeons. Among the possible causes, we figured the relative obsolescence of the spreader devices resulting nearly unchanged since 1941, when Finochietto developed his fortunate and long-lasting tool [13], only later mildly modified by Burford [14]. We hypothesized two main reasons determining rib fracture. The first one is due to the mismatch between the divaricating blade, usually straight or slightly bent, and the curvature of the rib constantly variating during the movement of opening. This divergence could create a point of major and sudden increment of pressure resulting in a force able to overtake the rib resistance. The second one might be the oscillating, nonlinear relationship between crank rotation and arm movement already described by Chanoit et al. [10]. This mechanism does not allow a gradual velocity control during spreading thus not giving time to thoracic tissues to relax and adapt and increasing the traumatic effect of the spreader. These are the reasons why we experienced in the majority of the cases the fractures of two adjacent ribs, bearing similar forces, pressures and accelerations.

The elevated number of rib fractures could be also reconducted to the choice of performing lateral thoracotomy, which could be more incline than postero-lateral one to cause this injury due to a possible increased counter-resistance to spreading given the presence of spared *latissimus dorsi.*

As expected, the analysis of factors correlated to rib fractures after thoracotomy revealed the age as the most significant one. The relationship was already described in low-energy traumas [15–17]. This predisposition should be likely correlated to the reduction of bone mineral density by age [15]. However, a real correlation between bone mineral density and traumatic rib fractures has been scientifically demonstrated only in female individuals after age-adjustment [16]. We did not experience a significant difference in the incidence of rib fractures for gender. This can confirm what was already described for traumatic rib fractures [16, 17].

We experienced that the presence of involuntary iatrogenic rib fractures developed during thoracotomy is associated with increased post-operative pain, especially when provoked by cough, rather than at rest. After a rib fracture, real pain is related to rib cage movements and consequently patients are more inclined to stay still, thus implying a worse and slower recovery with diaphragmatic dysfunction, ineffective cough and subsequent retention of bronchial secretions leading to increased risk of complications [18–21].

Thoracotomy is undoubtedly per se a painful incision regardless the extension of the cut. Jiwnani et al. showed no significant differences in post-operative pain between the standard posterolateral approach and a nerve-sparing one [22]. Nevertheless, there are no scientific evidence whether the presence of a rib fracture might determine a supplemental amount of discomfort. To the best of our knowledge our investigation is the first directly focused on the extent of this problem and its possible complications. Intriguingly, the presence of two adjacent rib fractures was not correlated to higher pain level compared to patients with single fracture, but this could be related to the consistent difference in the sample size of these two subgroups.

We are perfectly aware that this study has several limitations. Since its retrospective nature, we could have miscounted some rib fractures. In addition, the analysis of potential risk factors available for logistic regression was limited to the few ones described in the database. The evaluation of pain was limited only to the immediate post-operative period only due to the lack of long-term follow-up for most of the patients where having more information about chronic variations of the discomfort would have been more desirable, as chronic pain is one of the most important post-operative complications affecting the quality of life of patients after surgery.

## Conclusion

Thoracotomy is progressively becoming an infrequent procedure given the popularity of minimally invasive techniques. However, it is still necessary in case of complex surgeries or intra-operative complications. The required intercostal spreading might be cause of involuntary rib fracture. This event has not been adequately investigated so far. The present study demonstrated that rib fracture occurs in almost half of the thoracotomies. Older patients are more likely to develop this event, which resulted significantly correlated to increased evoked post-operative pain and higher rate of complications. These results may be a starting point for the improvement of post-operative management of these patients, like with the development of a specific and dedicated analgesia protocol and for the creation of a new less traumatic spreader.

## Data Availability

All data generated or analyzed during this study are included in this published article.
